# Botulinum toxin: a new differential diagnosis for a lytic bone lesion

**DOI:** 10.1186/s13256-024-04430-5

**Published:** 2024-03-24

**Authors:** Yael Lefkovits, Lara Lipton

**Affiliations:** 1 Department of Medical Oncology, Peter MacCallum Cancer Centre, Melbourne, Australia; 2 Department of Medical Oncology, Cabrini Hospital, Melbourne, Australia

**Keywords:** Bone cancer, Clinical oncology, Imaging, Medical oncology, Case report

## Abstract

**Background:**

Botulinum toxin, produced by the Gram-positive anaerobe *Clostridium botulinum*, is composed of seven antigenic subtypes (A, B, C, D, E, F, and G). Currently, only Botulinum toxin type A, commonly referred to as “Botox,” is approved for clinical use, given its relatively safe clinical profile. Botulinum toxin type A has a wide range of therapeutic indications, including treatment for dystonia, migraine headache, neurogenic bladder, and large muscle spastic disorders. However, the toxin is most widely known for its cosmetic effects in treating wrinkles and facial lines.

**Case presentation:**

This article describes a 62-year-old Caucasian female who presented for investigation and workup of an isolated lytic lesion of her frontal bone a few weeks after administration of botulinum toxin injection into the corresponding site in the frontalis muscle. This presented as a large, palpable, painless forehead lump causing significant psychological distress. After no neoplastic cause for the lesion was found and histopathology was performed, our researchers concluded that the most likely explanation was that the bony lytic lesion resulted from inadvertent injection of the “Botox” neurotoxin through the intended target muscle and into the cortex of the underlying bone.

**Conclusions:**

Our search of the literature failed to identify any previous cases of this occurring. However, as the popularity of this cosmetic procedure only increases, we believe that this represents an important possible differential for isolated lytic lesion after administration of “Botox” injection.

## Background

Botulinum toxin, produced by the Gram-positive anaerobe *Clostridium botulinum*, is composed of seven antigenic subtypes (A, B, C, D, E, F, and G) [1]. Currently, only Botulinum toxin type A (BTX-A), commonly referred to as “Botox,” is approved for clinical use, given its relatively safe clinical profile [2]. BTX-A has a wide range of therapeutic indications, including treatment for dystonia, migraine headache, neurogenic bladder, and large muscle spastic disorders [3]. However, the toxin is most widely known for its cosmetic effects in treating wrinkles and facial lines.

BTX-A chemodenervation is one of the most common cosmetic procedures performed worldwide [4]. Currently, Australians are estimated to spend over AUD $300 million per annum on these cosmetic injections [5]. In 2002, the FDA approved the use of BTX-A for cosmesis, specifically for “the temporary improvement in the appearance of moderate to severe glabellar lines associated with corrugator and/or procerus muscle activity in adult patients” [6]. The procedure is considered well tolerated with a broad safety margin [7]. However, as with any medical procedure, clinicians need to remain cognizant of the possible complications and adverse effects associated with paralytic neurotoxin injection.

We present the case of a 62-year-old Caucasian female who developed a protuberant mass at the site of a Botox injection to the forehead, a few weeks after administration of the agent. This presented as an isolated hard lump on her forehead evidenced on clinical examination. Imaging suggested that the mass was associated with an underlying osteolytic lesion of the frontal bone, and it is believed that inadvertent injection of BTX-A through the frontalis muscle and into the cortex of the underlying bone caused the lytic lesion. In animal studies, muscle paralysis has been associated with a loss of bone mineral density in the underlying bones, as it is postulated that reduced muscle force induces bone osteoclastic processes and disrupts bone homeostasis [8]. Although it is difficult to definitively attribute a causal association, this case provides important information of a possible additional significant risk associated with injection of BTX-A neurotoxin.

## Case presentation

A 62-year-old Caucasian woman presented to her general practitioner with 6-week history of a round, painless, fixed, hard mass overlying the right frontal bone. Her past medical history was significant for hypertension, managed with ramipril, and a previous cholecystectomy for cholecystitis. She had no significant mental health history. On examination, she was alert and orientated with normal vital signs and an unremarkable clinical examination except for this mass. Notably, she did not have organomegaly or lymphadenopathy and was otherwise well prior to the development of the mass. The woman had received an injection of 42 units of incobotulinumtoxin A (Xeoman^©^) into her face 6 weeks previously to reduce the appearance of forehead rhytides and glabellar lines and reported short-term pain after the injection. The injection was performed by a cosmetic physician who had not completed surgical training and was not a board-certified member of any formal Australian medical fellowship or college training program. The mass was located at the exact site of injection of the neurotoxin. This was the first time she had received the cosmetic injection. She had not had any other trauma to the area or recent falls.

A noncontrast brain computed tomography (CT) ordered by her general practitioner revealed a lytic defect within the right frontal bone measuring 2 × 1 cm^2^, with mild soft tissue swelling overlying it. A 1-cm hyperdense lesion was also noted within the pituitary fossa. No other abnormalities were detected. The lytic lesion itself was suspicious for a metastatic deposit. A subsequent bone scan confirmed the presence of a lytic lesion within the right frontal bone that demonstrated prominent increased activity. The suspected diagnosis at that stage was either plasmacytoma related to multiple myeloma or metastasis arising from a distant primary malignancy.

The patient was subsequently referred to an oncologist for further evaluation. She underwent bone scan, mammogram, and laboratory testing including a full blood examination, renal and liver function studies, thyroid studies, hormonal assays (FSH, LH, and prolactin), human growth hormone, and ACTH levels. All of these were within normal limits. Her tumor markers (CEA, CA125 and CA 19–9) were also within the normal range. She had no evidence of multiple myeloma on serum protein electrophoresis. The patient had no other significant medical history and was not taking any medications.

**Fig. 1:**
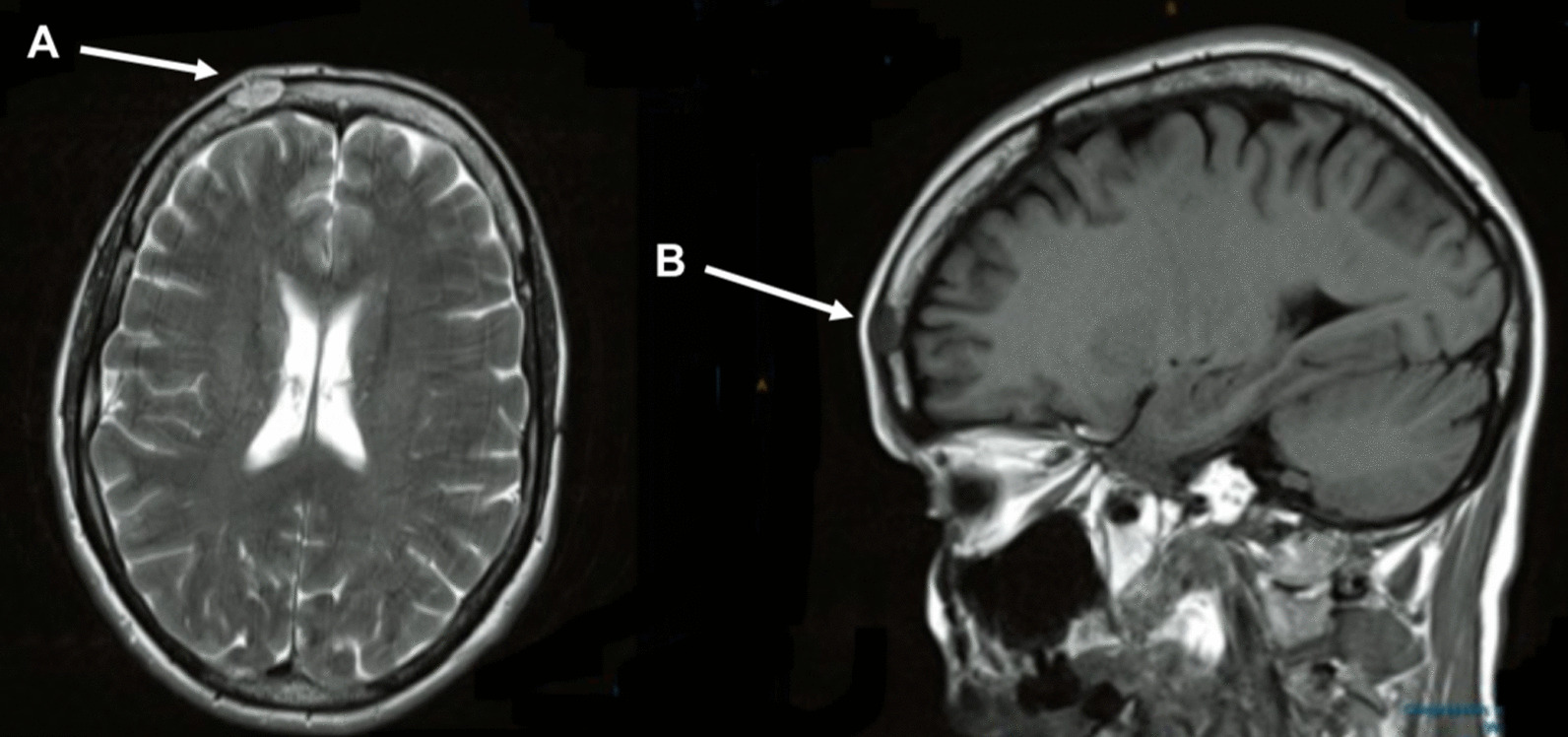
Head magnetic resonance imaging. **a** Axial T2 and **b** sagittal fluid-attenuated inversion recovery images. Both images demonstrate an oval 1.5-cm well-defined intradiploic lesion in the right frontal bone with a breach in the outer table of the skull overlying the lesion. The arrows denote the presence of the lytic lesion seen on magnetic resonance imaging

She subsequently underwent an MRI scan, which as seen in Fig. 1, demonstrated minor chronic deep white-matter ischemic changes with no intraaxial mass lesion or leptomeningeal enhancement. A 1.5-cm soft tissue mass was noted in the right frontal bone with a probable break in the cortex anteriorly. There was no epidural mass, and the inner table was preserved. This appearance was considered abnormal but nonspecific. In addition, a 0.9-cm nonenhancing hyperintense lesion in the right suprasellar cistern was noted. This appearance was most in keeping with a Rathke’s pouch cyst and thought to be unrelated to the lytic lesion.

Our patient’s images did not exhibit any characteristic features seen with known benign causes of osteolytic skull lesions. An arachnoid granulation would likely have appeared as a well-defined round lesion on CT, protruding into the calvarium; epidermoid cysts tend to present as nonenhancing hypodense focal lesions with calcification [9, 10], while intraosseous hemangiomas are usually characterized by an expansive lesion with thin margins and intralesional spicules radiating from a common center [9, 11]. Eosinophilic granulomas—a benign form of Langerhans cell histiocytosis—often contain a small sequestrum of devascularized bone, providing a “bull’s eye” appearance on MRI, and are associated with localized pain, mild peripheral eosinophilia, and fever [12]. Aneurysmal bone cysts, usually located in the long-bone epiphyses, often show osteoclast-like multinucleated giant cells expressing activator of nuclear kappa B receptors or neoplastic stromal cells expressing the receptor activator of nuclear factor kappa-Β (RANK) ligand [13, 14].

**Fig. 2:**
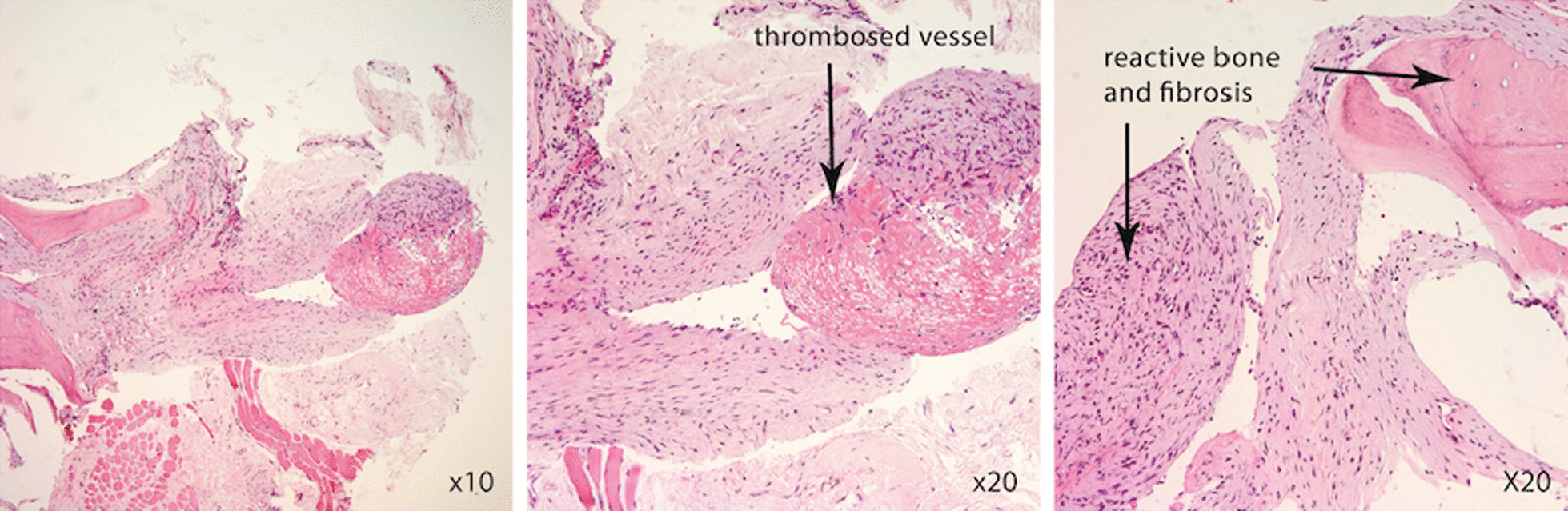
Hematoxylin and eosin-stained section from the core biopsy, demonstrating reactive bone with intertrabecular fibrosis and small vessel proliferation. There is a dilated blood vessel containing a fibrin thrombosis and small strips of skeletal muscle fibers. Magnification ×200, scale bar 100 μm. The arrows denote the presence of the lytic lesion seen on magnetic resonance imaging

An ultrasound-guided biopsy of the lesion was performed. The fine-needle aspirate yielded 8 ml of bloody fluid. On light microscopy, the aspirate contained blood, proteinaceous material, a mildly increased number of neutrophils, and a few lymphocytes. There were no plasma cells or cytological evidence of malignancy. On histopathology, sections from the core biopsy showed reactive bone associated with intertrabecular fibrosis and small-vessel proliferation. There was a dilated blood vessel containing a fibrin thrombosis, and small strips of skeletal muscle fibers were seen, as seen in Fig. 2. No evidence of malignancy was detected. Her case was presented by her oncologist at a weekly multidisciplinary meeting and discussed with specialist pathologists, radiologists, orthopedic surgeons, a plastic surgeon, and medical and radiation oncologists. It was concluded that the lytic lesion was benign, likely related to the injection of Botox 6 weeks earlier.

Unfortunately, she was advised by numerous plastic and orthopedic surgeons that there were no surgeries or cosmetic procedures available that would yield a favorable cosmetic outcome to improve the appearance of the mass. She was advised that no further follow-up was required and she did not require any follow-up imaging. On review 1 year later with her general practitioner, the lesion was still present with negligible improvement, which was a source of significant distress for her. She had sought out ultrasound-guided steroid injections and acupuncture to attempt to improve the appearance of the mass but with no noticeable effect. She was referred to a psychologist for ongoing psychological support.

## Discussion and conclusion

To our knowledge, this is the first report of the formation of a substantial bony lytic lesion that likely resulted from inadvertent injection of “Botox” neurotoxin through the intended target muscle into the cortex of the underlying bone. We believe that there are a number of clinical factors that support this conclusion. Our patient was clinically well, with no evidence of primary or secondary malignant disease. The lesion was in the exact location of the previous injection site, and MRI demonstrated a probable break in the cortex anteriorly—conceivably due to unintended insertion of the needle through the frontalis muscle into the periosteum. There were no features to suggest an alternative diagnosis for the osteolytic lesion, and the histopathology showed reactive bone formation, consistent with injury or irritation to the bone.

Botox is generally considered to have an excellent clinical safety profile, with most adverse effects being minor and transient [15]. The botulinum toxin is produced by the bacterium *Clostridium botulinium* [7]. After injection, the toxin diffuses into the tissue and reversibly binds to the presynaptic terminal of the neuromuscular junction, before attaching to a protein membrane responsible for acetylcholine excretion. The neuromuscular junction is inhibited from releasing acetylcholine, which causes a reversible relaxation of the facial muscles, thus reducing the appearance of wrinkles and fine lines [16, 17].

Adverse effects are usually local and include erythema, ecchymoses, and pain around the injection site [6]. The specific adverse effect of Botox forming a lytic lesion in bone has not been reported in humans. However, in an animal study, rats treated with BTX-A into the temporalis and masseter muscles to induce masticatory hypoactivity showed a reduced cortical bone thickness and bone mineral density (BMD) of the skull [18]. A further animal model study analyzed the effects of botulinum toxin A injections into the masseter muscle and demonstrated a negative effect on bone growth [8]. The authors postulated that paralysis of the overlying muscle can affect bone remodeling by inducing bone osteoclastic processes and contributing to bone degradation [8]. This is based on the functional matrix theory that the overlying soft tissue function and muscle activity are major contributors to bone growth and that paralysis of the overlying muscle induces bone loss through reduction in bone mineral content [8]. Other animal studies have demonstrated an increase in trabecular and cortical bone loss when overlying muscles were temporarily paralyzed by the neurotoxin [19, 20]. Whether these effects were a function of the resultant muscular hypoactivity or an indirect osteolytic effect remains uncertain.

With the increasing popularity of Botox, for both cosmetic and therapeutic indications [5], it is likely that this type of sequelae after administration of such injections will become more commonplace [5]. Overall, the use of Botox injections to treat hyperkinetic facial lines is considered to be a reasonably safe procedure. However, severe complications such as the one reported herein serve as a warning of the potential dangers, the importance of adequate training for administrators, and the need for appropriate monitoring of patient outcomes and adverse events.

## Data Availability

The dataset supporting the conclusions of this article are included within the article.

## Abbreviations

FDA: Food and Drug Administration
BW: Body weight
FSH: Follicle stimulating hormone
LH: Luteinizing hormone
ACTH: Adrenocorticotropic hormone
CEA: Carcinoembryonic antigen
CA 125: Cancer antigen 125
CA 19–9: Cancer antigen 19–9
MRI: Magnetic resonance imaging
